# Complete Genome Sequence of *Staphylococcus aureus* Z172, a Vancomycin-Intermediate and Daptomycin-Nonsusceptible Methicillin-Resistant Strain Isolated in Taiwan

**DOI:** 10.1128/genomeA.01011-13

**Published:** 2013-12-05

**Authors:** Feng-Jui Chen, Tsai-Ling Lauderdale, Lih-Shinn Wang, I-Wen Huang

**Affiliations:** National Institute of Infectious Diseases and Vaccinology, National Health Research Institutes, Zhunan, Taiwana; Department of Internal Medicine, Division of Infectious Diseases, Tzu Chi General Hospital, Hualien, Taiwanb

## Abstract

We report the complete genome sequence of Z172, a representative strain of sequence type 239-staphylococcal cassette chromosome *mec* type III (ST239-SCC*mec* type III) hospital-associated methicillin-resistant *Staphylococcus aureus* in Taiwan. Strain Z172 also exhibits a vancomycin-intermediate and daptomycin-nonsusceptible phenotype.

## GENOME ANNOUNCEMENT

Vancomycin is the last line of defense against serious methicillin-resistant *Staphylococcus aureus* (MRSA) infections. The emergence of vancomycin nonsusceptibility in MRSA adds to the challenge of limited treatment choices (1, 2). A number of chromosomal factors have been found to influence vancomycin heterogeneous resistance in *S. aureus* (3). To further facilitate investigations on the genetic changes associated with vancomycin-intermediate *S. aureus* (VISA), we selected one VISA strain (vancomycin MIC, 4 µg/ml) for genome sequencing. This strain also exhibits a daptomycin-nonsusceptible phenotype (MIC, >4 µg/ml). Here, we report the complete genome sequence of MRSA Z172, a sequence type 239-staphylococcal cassette chromosome *mec* type III (ST239-SCC*mec* III) strain isolated in 2010 from a blood specimen of an elderly intensive care unit (ICU) patient during part of the Taiwan Surveillance of Antimicrobial Resistance (TSAR) project (4). The ST239-SCC*mec* III MRSA clone type is the most prevalent group of hospital-associated MRSA (H-MRSA) strains in Taiwan belonging to clonal complex 8 (CC8) (5).

The whole genome of strain Z172 was sequenced using a combination approach of Illumina/Solexa sequencing with a 200-bp paired-end library and PacBio sequencing with a 10-kb library, which generated 26,067,268 reads (about 899-fold genome coverage) and 49,306 reads (about 77-fold genome coverage), respectively. The resulting sequences were assembled using the PacBioToCA pipeline in the Celera assembler 7.0 (6, 7). The sequence gaps were closed using the CLC Genomics Workbench version 6.5 (CLC bio, Aarhus, Denmark). The assembled genome was validated by the Argus optical mapping system with AflII digestion (OpGen, Madison, WI). The complete genome of *S. aureus* strain Z172 contains a circular chromosome of 2,987,966 bp (32.84% G+C content) and two circular plasmids of 27,326 bp and 3,011 bp in size. Sequence annotations of the chromosome and plasmids were performed by Rapid Annotations using Subsystems Technology (RAST) (8), followed by manual inspection and comparisons with other *S. aureus* genomes. A total of 2,834 predicted coding sequences (CDSs) were detected on the chromosome. In addition to the CDSs, RAST revealed 76 RNA genes, including 60 tRNA and 16 rRNA genes.

There are three published genomes of *S. aureus* strains with the ST239 sequence type, JKD6008, TW20, and T0131, which were isolated in New Zealand (9), England (10), and China (11), respectively. A comparison with these three published *S. aureus* genomes revealed that Z172 contains three prophages, a 47.7-kb φSa1, a 50-kb φSa3, and 62.6-kb φSPβ-like prophage. However, only strain TW20 contains a φSa1(TW20) prophage, but its location differed from that of φSa1(Z172). In addition, the φSPβ-like prophage was found only in TW20 and Z172. However, φSPβ-like(Z172) only contains the proximal half sequence of φSPβ-like(TW20). Besides these prophages, Z172 also has two plasmids (pZ172_1 and pZ172_2), as is the case with TW20 (pTW20_1 and pTW20_2). The sequences of the large plasmids pZ172_1 and pTW20_1 are nearly identical except for Tn*552*, which is absent in pZ172_1. The sequences of the smaller plasmids, pZ172_2 and pTW20_2, are identical. These plasmids are absent in JKD6008 and T0131. The finding that Z172 and TW20, which are from distant regions, share the highest genome similarity suggests that a successful intercontinental transmission event may have occurred.

### Nucleotide sequence accession numbers.

The whole-genome sequence of *S. aureus* Z172 has been deposited in the DDBJ/EMBL/GenBank databases under accession no. CP006838, CP006839, and CP006840. The version described in this paper is the first version.
